# The Relationship between Human Embryo Parameters and De Novo Chromosomal Abnormalities in Preimplantation Genetic Testing Cycles

**DOI:** 10.1155/2022/9707081

**Published:** 2022-03-19

**Authors:** Yanli Liu, Junhan Shen, Rujing Yang, Yuchao Zhang, Liting Jia, Yichun Guan

**Affiliations:** ^1^Department of Reproductive Medicine, The Third Affiliated Hospital of Zhengzhou University, Zhengzhou, Henan, China; ^2^Neonatal Screening Center, The Third Affiliated Hospital of Zhengzhou University, Zhengzhou, Henan, China

## Abstract

**Design:**

In total, 456 PGT cycles, including 283 PGT-SR cycles and 173 PGT-A cycles, were assessed through comprehensive chromosome screening (CCS) from January 2017 to June 2020 at the Department of Reproductive Medicine of the Third Affiliated Hospital of Zhengzhou University. Trophectoderm (TE) biopsies were sequenced using next-generation sequencing (NGS). The incidence of de novo chromosome abnormalities was calculated, and the relationships between de novo chromosome abnormality rates and maternal age, number of oocytes retrieved, and parameters of cleavage-stage embryos and blastocyst-stage embryos were investigated.

**Results:**

The incidence of de novo chromosome abnormalities was 28.0% (318/1,135) in the PGT-SR cycles and 36.3% (214/590) in the PGT-A cycles, which increased with maternal age in both PGT-SR cycles (*P* = 0.018) and PGT-A cycles (*P* < 0.001). The incidence of de novo chromosome abnormalities was related to TE grade (*P* < 0.001), internal cell mass grade (*P* = 0.002), and development speed (day 5 vs. day 7: *P* < 0.001) of blastocyst-stage embryos. The incidence of de novo chromosomal abnormalities was irrelevant to the number of oocytes retrieved and the parameters of the embryo at the cleavage stage.

**Conclusion:**

Blastocysts with higher morphology scores and faster progression had a lower incidence of de novo chromosome abnormalities, especially complex chromosome abnormalities. De novo chromosome abnormalities may negatively affect the morphological grading of blastocysts. Our findings will provide valuable information to the fertility doctor for embryo selection in non-PGT cycles.

## 1. Introduction

As the incidence of infertility has increased continuously in recent years, an increasing number of couples are turning to assisted reproductive technology for fertility treatment [1, 2]. The goal of assisted reproductive technology is to select embryos with maximum potential for transfer and obtain a single healthy live pregnancy in the shortest time. However, the incidence of chromosomal abnormalities in early human embryos cultured in vitro is high up to 60% [3, 4], and this is strongly associated with implantation failure and abortion [5]. Preimplantation genetic testing (PGT), including testing for structural rearrangements (PGT-SR), aneuploidy (PGT-A), and monogenetic disorders (PGT-M), is an effective method of identifying chromosome abnormalities in early embryos. It can prevent the transmission of pathogenic genetic mutations or chromosomal aberrations [6, 7].

In PGT cycles, some aneuploidies are inherited from either parental carrier of chromosome abnormalities [8], and these are called genetic abnormalities [9]. Alternatively, genetic abnormalities can be generated de novo and are termed “de novo chromosome abnormalities” [10]. This may be caused by mitotic errors during embryonic development or meiotic errors during gametogenesis [9]. In PGT-A cycles, aneuploidy is considered to be de novo chromosome abnormality, as both parents have a normal chromosomal karyotype. Chromosomal abnormalities, whether inherited or arising de novo, can be transmitted from fertilization to conceptus. This is an important factor causing embryo arrest, implantation failure, or miscarriage and may cause adverse perinatal outcomes such as stillbirth, neonatal death, birth defects, and intellectual impairments [11, 12].

Some studies on de novo chromosome abnormalities have focused on the prenatal diagnosis involving fetal components such as amniotic fluid, the chorionic villus, or fetal blood in the prenatal diagnosis [13, 14] or on the incidence of newborns carrying de novo chromosome abnormalities [15, 16]. Others have examined the relationship between the incidence of de novo chromosome abnormalities and the month of conception [17] or the incidence of de novo segmental aneuploidy in PGT-A cycles [18].

Fewer studies have focused on cycles involving PGT  of de novo mutations. Magli et al. [19] analyzed the incidence of de novo segmental aneuploidy (SA) in oocytes, cleavage-stage embryos, and blastocysts in couples with normal and balanced chromosome rearrangements and detected the highest incidence in cleavage-stage embryos. Zhou et al. [17] found that the incidence of de novo segmental aneuploidies is not related to maternal age and that they can occur on all chromosomes. Other researchers [20, 21] studied the relationship between embryo morphology and ploidy status in PGT-A cycles, with parameters including cell number and embryo fragmentation at the cleavage stage, trophectoderm (TE) morphology, inner cell mass (ICM) morphology, blastocyst expansion, and day of TE biopsy at the blastocyst stage. The studies found that a better TE morphology grade was associated with a higher euploidy rate, while embryo morphology at the cleavage stage was not relevant to ploidy status.

However, to the best of our knowledge, this is the first systematic evaluation and theoretical verification comparison of the relationship between de novo chromosome abnormalities and embryo morphological parameters in both PGT-A and PGT-SR cycles. The aim of our study was to evaluate possible relationships among maternal age, number of oocytes retrieved, and embryo morphological parameters with de novo chromosome abnormalities, as this may assist in selecting embryos with a low risk of de novo chromosome abnormalities for non-PGT cycles.

## 2. Materials and Method

### 2.1. Patients

This retrospective study was performed at the IVF Center of Reproductive Medicine of the Third Affiliated Hospital of Zhengzhou University in China from January 2017 to June 2020. Patients were included in the treatment of PGT-SR and PGT-A. A total of 283 cycles underwent PGT-SR for reciprocal translocation (REC), Robertsonian translocation (ROB), or inversion (INV) observed in at least one member of the couple. A total of 173 cycles underwent PGT-A because of advanced maternal age, recurrent miscarriage, or repetitive implantation failure, although the parents had normal chromosomal karyotypes. De novo chromosome abnormalities were defined as losses or gains larger than 4 Mb in size that were not inherited from either of the subject's parents. De novo chromosome abnormalities were classified into subchromosome (segmental) abnormalities, whole-chromosome abnormalities, and complex chromosome abnormalities [22]. The study was conducted in accordance with the Code of Ethics in the Declaration of Helsinki and approved by the ethics committee of the Third Affiliated Hospital of Zhengzhou University. As the study was retrospective and patient data were analyzed anonymously, no informed consent was required.

### 2.2. Controlled Ovarian Stimulation, Oocyte Retrieval, and Fertilization

All patients in our study had an ultrasound scan and a serum evaluation of anti-Müllerian hormone (AMH) and sex hormone levels on the third day of the menstrual cycle, and these parameters were used to evaluate their ovarian reserve function. Ovarian stimulation was performed with a gonadotropin-releasing hormone (GnRH) antagonist protocol, GnRH agonist protocol, or mild stimulation protocol. Human chorionic gonadotropin (hCG) was administered to promote oocyte maturation when two or more leading follicles reached a diameter of more than 18 mm, and transvaginal ultrasonography-assisted oocyte retrieval was performed 36 h after the hCG injection. Then, the oocyte-corona-cumulus complex (OCCC) was cultured for 2 h before the removal of granulosa cells. To avoid result deviations, granulosa cells were removed completely. Intracytoplasmic sperm injection (ICSI) was performed only for MII oocytes. If the oocytes were at the MI or GV stage, they were cultured in vitro until maturation for another 24 h and then fertilized until maturity.

### 2.3. Embryo Culture and Trophectoderm Biopsy

The oocytes were placed in G-1 medium (Vitrolife) in an incubator with 5% O_2_, 6% CO_2_, and 89% N_2_ after fertilization until day 3. All day 3 embryos were transferred to G-2 medium (Vitrolife) to be cultured for another 3-4 days. Zygotes were observed 16–18 h after fertilization. Only 2PN-derived embryos were included in this study. Embryos were observed and scored according to the Istanbul consensus on day 2 and day 3 [23]. The morphology scoring system was based mainly on cell number and fragmentation. Blastocysts were scored according to Gardner and the Schoolcraft system [24]. At our center, blastocysts with a score above 3 BC were defined as available blastocysts. Approximately 5–10 TE cells were biopsied using the laser-assisted method from available and expanded blastocysts on days 5–7 after insemination. The blastocysts were vitrified after they were biopsied.

### 2.4. Sample Preparation and NGS Analysis

Each biopsied cell mass was carefully washed in G-MOPS Plus (Vitrolife) to remove any DNA contaminant first and then transferred into a 0.2 ml polymerase chain reaction (PCR) tube that contained 2 *μ*l PBS, pulse-centrifuged quickly, and stored at −20°C until analysis. DNA was extracted from the biopsy samples and then amplified using a Sure Plex Single-Cell WGA kit. An Ion Plus Fragment kit (Life) was used to prepare the NGS libraries. Unique adapter sequences at the ends of the fragments were used to amplify the insert DNA, and specific sequences were added to complete sequencing by limited-cycle PCR. The Ion PGM Hi-Q OT2 Kit-200 (Life) was used to amplify and enrich the library. An Ion PGM Hi-Q sequencing kit (Life) was used to perform 260 flow-read sequencing procedures according to the library construction formula. The PGX Cloud platform was used for vehicle-mounted auxiliary data analysis. Detection and classification of aneuploidies were determined based on copy number variation (CNV) values. Euploidy was considered when CNV values were from 1.80 to 2.20. Aneuploidy was categorized as pure when CNV values were <1.20 or >2.80. Aneuploidies were considered to be mosaic when CNV values were from 1.20 to 1.80 or from 2.20 to 2.80. De novo chromosome abnormalities were determined by whether they were inherited from either of the parents. Importantly, embryos with euploidy, mosaic aneuploidy, or parent-inherited aneuploidy were classified as being without de novo chromosome abnormalities in the PGT-SR cycles, while embryos without euploidy or mosaic aneuploidy were classified as having de novo chromosome abnormalities in the PGT-A cycles.

### 2.5. Definition and Statistical Analysis

Embryos with one or more whole-chromosome abnormalities were classified as having whole-chromosome abnormalities; embryos with one or more segmental-chromosome abnormalities were classified as having segmental-chromosome abnormalities; and embryos with at least one whole-chromosome abnormality and at least one segmental-chromosome abnormality were classified as having complex abnormalities.

Categorical variables are presented as *n* (%), and continuous variables are presented as the mean ± SD. The chi-square test or Fisher's exact test was used for categorical variables, and the relationship between the incidence of de novo chromosome abnormalities and embryo parameters was analyzed using logistic regression. *P* value was calculated using the mixed logistic model adjusted by female age and PGT-SR/PGT-A scheme. *P* < 0.05 was considered statistically significant.

## 3. Results

### 3.1. Incidence of De Novo Chromosome Abnormalities in Preimplantation Embryos

A total of 456 cycles including 1,764 blastocysts were included in this study. The number of cases, demographic parameters of all cycles, and results of genetic testing of blastocysts are presented in Table 1. Among the blastocysts, the rate of aneuploid blastocysts was significantly higher in PGT-SR cycles than that in PGT-A cycles (36.3%), and the rate of de novo chromosome abnormalities in PGT-SR cycles (28.0%) was lower than that in PGT-A cycles (36.3%). De novo segmental chromosomes were the most common type in PGT-SR cycles, and de novo whole chromosomes were the most common type in PGT-A cycles (Table 1). The proportion of de novo whole chromosomes (21.2%) in PGT-A cycles was significantly higher than that in PGT-SR cycles (9.9%) (*P* < 0.01); there was also a significant difference in the de novo segmental chromosomes in PGT-A and PGT-SR cycles (9.0% and 12.6%).

### 3.2. Maternal Age and De Novo Chromosome Abnormality

To evaluate whether the incidence of de novo abnormalities was related to maternal age, patients were divided into different groups. As presented in Table 2, the incidence of de novo chromosome abnormalities was lowest among patients younger than 29 years (24.0%) and highest among patients older than 40 years (64.9%). The incidence of blastocysts with de novo chromosome abnormalities rose steadily with age. There were significant differences among the different age groups of both the PGT-SR (*P* = 0.018) and PGT-A (*P* < 0.001) groups.

### 3.3. Number of Retrieved Oocytes and De Novo Chromosome Abnormalities

As shown in Table 3, there was no significant difference in the incidence of de novo chromosome abnormalities with different numbers of oocytes retrieved (*P* = 0.073). In addition, no significant difference in the incidence of de novo chromosome abnormalities was found for a maternal age over 35 years (*P* = 0.578) or under 35 years (*P* = 0.923) in subgroups with different numbers of oocytes retrieved.

### 3.4. Embryo Parameters and De Novo Chromosome Abnormality

The incidence of de novo chromosome abnormalities was related to ICM grade (ICM A vs. ICM B; *P* = 0.002), TE grade (*P* < 0.001), and day of blastocyst biopsy (day 5 vs. day 7; *P* < 0.001) in PGT cycles (Table 4). Blastocysts with higher ICM grades, higher TE grades, and a faster development speed had a significantly lower incidence of chromosome abnormalities. No correlation was found between the expansion of blastocysts and the incidence of de novo chromosome abnormalities. In addition, the incidence of de novo chromosome abnormalities was not related to the parameters of cleavage-stage embryos, including cell number or fragmentation.

### 3.5. The ICM Grade, TE Grade, and Biopsy Day of the Blastocyst Related to the Type of De Novo Chromosome Abnormality

**Figure 1:**
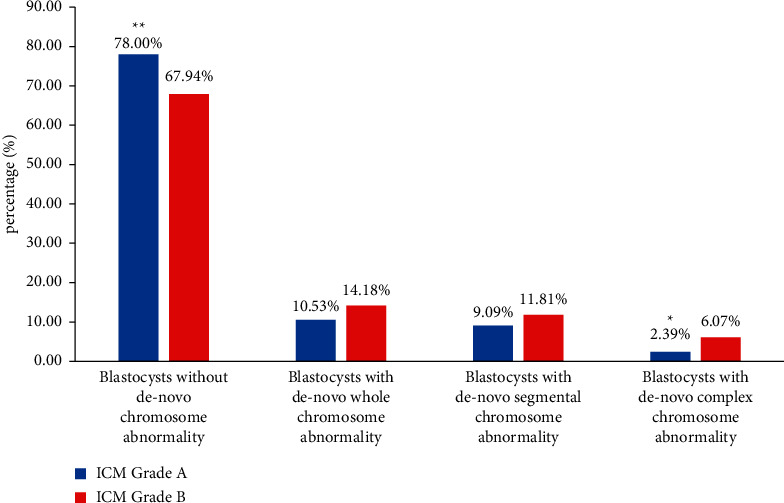
The proportion of blastocysts with different genetic test results related to ICM quality. Data were presented as a percentage. Blastocysts without de novo chromosome abnormality contained euploid, mosaic and chromosome abnormalities inherited from either parent. The proportions between the two groups were tested by the chi-square test.  ^*∗∗*^*P* < 0.01 vs ICM grade B and  ^*∗*^*P* < 0.05 vs ICM grade B.

**Figure 2:**
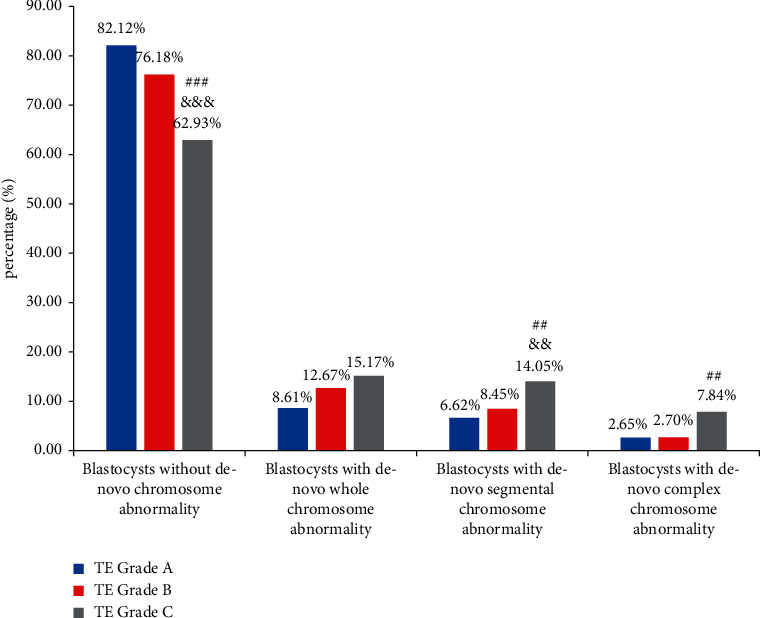
The proportion of blastocysts with different genetic test results related to TE quality. Data were presented as a percentage. Blastocysts without de novo chromosome abnormality contained euploid, mosaic and chromosome abnormalities inherited from either parent. The proportions between the three groups were tested by the chi-square test. ^###^*P* < 0.001 vs TE grade B, ^&&&^*P* < 0.001 vs TE grade A, ^##^*P* < 0.01 vs TE grade B, and ^&&^*P* < 0.01 vs TE grade A.

**Figure 3:**
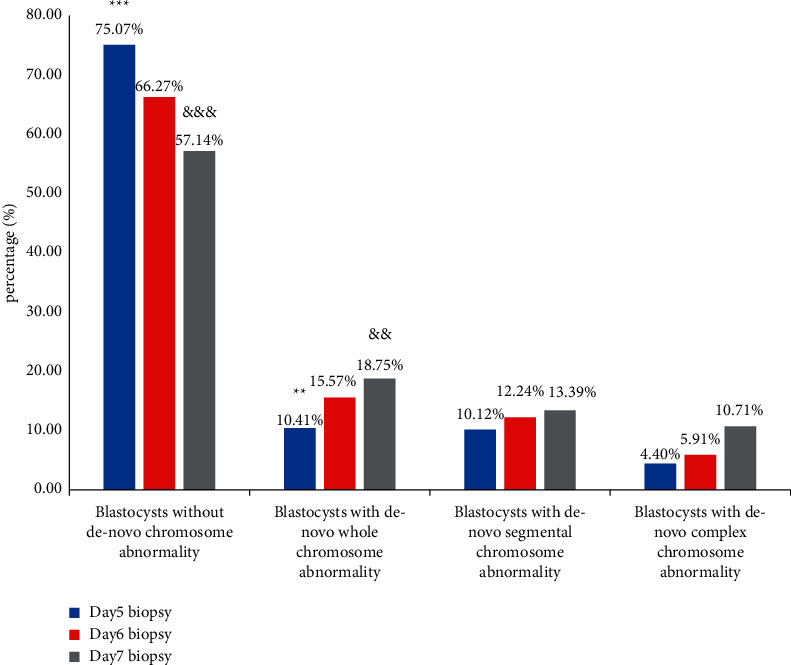
The proportion of blastocysts with different genetic test results related to the biopsied day. Data were presented as a percentage. Blastocysts without de novo chromosome abnormality contained euploid, mosaic and chromosome abnormalities inherited from either parent. The proportions between the three groups were tested by the chi-square test.  ^*∗∗∗*^*P* < 0.001 vs biopsied on D6, ^&&&^*P* < 0.001 vs biopsied on D5,  ^*∗∗*^*P* < 0.01 vs biopsied on D6, and ^&&^*P* < 0.01 vs biopsied on D5.

To further elucidate the relationship between blastocyst parameters and the type of de novo chromosome abnormalities, de novo segmental abnormalities, de novo whole-chromosome abnormalities, and de novo complex chromosome abnormalities were identified. As shown in Figure 1, the incidence of complex de novo chromosome abnormalities in ICM grade A embryos was lower than that in ICM grade B embryos (*P* < 0.05). There was a significant difference in the incidence of de novo segmental-chromosome and complex chromosome abnormalities for blastocysts with different TE grades. A higher incidence of de novo chromosome abnormalities was more strongly associated with TE grade C than TE grade A or B (segmental: grade A vs. grade C, *P* < 0.01; segmental: grade B vs. grade C, *P* < 0.01; complex: grade A vs. grade C, *P* < 0.01; Figure 2). Biopsy day was related to the incidence of whole de novo chromosome abnormalities (day 5 vs. day 6, *P* < 0.01; day 5 vs. day 7, *P* < 0.01) (Figure 3). Blastocysts with faster development had a lower incidence of whole de novo chromosome abnormalities.

## 4. Discussion

The study involved 456 PGT cycles from 407 couples. The incidence of de novo chromosome abnormalities was 28.0% in the PGT-SR cycles and 36.3% in the PGT-A cycles, and these rates increased with maternal age. As morphological evaluation remains the main strategy used to select embryos for transfer in most reproductive centers, we analyzed the relationship between the incidence of de novo chromosome abnormalities and embryo morphology. We observed that the parameters of blastocysts were related to de novo chromosome abnormalities, and blastocysts with poor ICM and TE quality or slow development were more likely to have a higher probability of de novo chromosome abnormalities. This is one of the main findings of our study, and this finding provides potential guidance for embryo selection. Additionally, there was no correlation between the incidence of de novo chromosome abnormalities, the number of oocytes retrieved, or the parameters of cleaved embryos. To our knowledge, this was the first study to examine the relationship between maternal age, oocyte number, embryo parameters, and de novo chromosome abnormalities in PGT cycles.

The accuracy of the embryo biopsy stage and the chromosome screening method is critical for PGT technology [25]. In blastocyst-stage TE biopsy, a smaller percentage of the embryo is removed without affecting embryonic implantation potential [25]. Moreover, 5–10 cells can provide more DNA, decreasing the probability of amplification failure and technical bias amplification or allele drop out (ADO) [26]. This can minimize the risk of mosaic and ensure the accuracy of the results. Next-generation sequencing (NGS) is an effective technique for analyzing CNV in single cells and can avoid the shortcomings of the limited probes in fluorescent *in situ* hybridization (FISH) technology and low-throughput PCR technology. All 23 pairs of chromosomes could be tested at a high resolution. What matters most is that NGS can detect whole-chromosome aneuploidies and segmental aneuploidies in single blastomeres [9, 27]. Furthermore, it can reduce costs and be highly reproducible [28].

Among patients undergoing PGT-SR cycles, it was typical that at least one member of the couple had structural abnormalities, such as REC, ROB, or INV. Owing to the structural rearrangement of chromosome translocation carriers, the separation of chromosomes other than translocated chromosomes may be affected during the formation of gametes. This phenomenon is called interchromosome effect (ICE) and is caused by genomic instability and abnormal chromosome separation [29], which may result in the incidence of de novo chromosome abnormalities being higher in PGT-SR cycles than in PGT-A cycles. However, our study showed the opposite results. The incidence of de novo chromosome abnormalities in PGT-SR cycles (28.0%) was lower than that in PGT-A cycles (36.3%), which may be due to the infertility factors of patients and sample size in PGT-A cycles.

It is well known that the incidence of embryo aneuploidy increases rapidly with maternal age, and previous studies have provided abundant evidence [4, 30, 31]. In our study, de novo chromosome abnormalities were equal to aneuploidy in PGT-A cycles, as we mentioned before. We found the same trend: the incidence of de novo chromosome abnormalities increased with advanced maternal age in both PGT-A cycles and PGT-SR cycles. It was 24.0% for patients younger than 29 years and 64.9% for patients older than 40 years in the PGT cycles (Table 2). This may be due to chromosome abnormalities in oocytes caused by maternal meiosis errors [32], which were positively associated with increased maternal age [33]. This view is supported by other scholars. For example, Chiang et al. found that recombination errors associated with checkpoints in meiosis I and sister chromatid cohesion deterioration increased with age in early meiosis [34]. Hook et al. found that the production of de novo marker chromosomes was related to the advanced age of the pregnant woman [35].

The aim of ovarian stimulation is to recruit more follicles to enable the acquisition of more oocytes, which increases the probability of obtaining euploid embryos. However, the influence of a high number of oocytes on embryo ploidy is in dispute [36, 37]. Some scholars suggest that the decline in the number of oocytes retrieved is associated with an increase in euploidy [36]. Presumably, high doses of Gn or a high response to stimulation might be embryotoxic and increase aneuploidy rates or de novo chromosome abnormalities [38]. In our study, de novo chromosome abnormalities were not associated with different numbers of oocytes retrieved for the ≤5-oocyte, 6–10-oocyte, 11–15-oocyte, or ≥16-oocyte subgroup. It was concluded that abundant oocyte yield was not embryotoxic and that neither high doses of Gn nor a strong response to Gn increases the abnormal segregation of chromosomes during meiosis [39]. Some researchers hold similar opinions. Euploidy rates for women of different ages are comparable between groups when different numbers of oocytes are retrieved [40, 41].

In this study, the relationship between de novo chromosome abnormalities and embryonic parameters was of great concern. We selected 2PN-derived zygotes and analyzed the relationship between embryonic parameters and de novo chromosome abnormalities based on the following factors: parameters of cleavage-stage embryos including the cell number and embryo fragmentation on days 2 and 3; parameters of embryos at the blastocyst stage including development speed, ICM, and TE grades; and blastocyst expansion. Our results indicated that the parameters of cleavage-stage embryos are irrelevant to the incidence of de novo chromosome abnormalities. The high-grade morphological evaluation did not assist in the reduction of meiotic errors [42, 43]. Similarly, previous studies showed that the morphology of the cleavage-stage embryos did not affect ploidy [21, 44]. The study confirmed that de novo chromosome abnormalities had no effect on the morphologic scores of embryos at the cleavage stage. The association between the morphological evaluation of cleavage-stage embryos and chromosome status remains weak.

De novo chromosome abnormalities were found to be related to the ICM grade and the developmental speed of blastocysts and especially to the trophoblast cell grade. Similar to previous studies [20, 45], we found increased de novo chromosome abnormality occurrence among blastocysts with lower TE and ICM grades (Table 4). Although the underlying mechanism was not clear, it could be inferred that de novo chromosome abnormalities have a negative effect on the TE and ICM grades, leading to blastocyst morphological decline and developmental arrest [5, 10]. A high-quality ICM and TE are critical for facilitating normal embryogenesis and development. A poor-quality TE and ICM are potential indicators of chromosomal aberrations in embryos [46]. Therefore, TE grade A and ICM grade A are associated with better implantation potential and a lower risk of miscarriage than grades B and C [47, 48]. Another parameter analyzed in the study was the time for embryos to develop into expanded blastocysts. Blastocysts biopsied on day 5 showed a significantly lower risk of de novo chromosome abnormalities than those biopsied on day 7, while blastocysts biopsied on day 6 presented a similar risk of de novo chromosome abnormalities to that of blastocysts biopsied on day 7. The rate of blastocyst formation was associated with the incidence of de novo chromosome abnormalities. It was speculated that delayed blastulation was affected by de novo chromosome abnormalities. This finding was supported by other researchers: Hernandez-Nieto et al. [49] found that the euploidy rate was significantly lower on day 7 than on day 5 or day 6 (40.5% vs. 54.7% vs. 52.9%, respectively); Kaing et al. [50] also thought that earlier blastocyst development was associated with a significantly increased euploid rate. However, Alfarawati et al. showed that the correlation between blastocyst morphology and aneuploidy remains weak in PGT-A cycles [51]. This is probably because zona opening in cleavage-stage embryos interfered with embryo development from the cleavage stage to the blastocyst stage, which lowered the reliability of the results compared with the zona pellucida performed at the blastocyst stage.

The relationship between the different types of de novo chromosome abnormalities and blastocyst morphological grades was assessed in a secondary analysis. SA accounted for ∼6% of spontaneous abortions [52]. Using microarray technology, Vanneste et al. also found that ∼70% of cleavage-stage embryos had at least one cell that was affected by segmental imbalance [53]. Segmental abnormalities originate from error correction of chromosome breakage, which may be caused by chromosome instability, or are introduced by ovulation induction and the biopsy procedure [54, 55]. Recently, Vera-Rodríguez et al. investigated whether the origin of mitosis is the cause of embryonic segment aneuploidy at the blastocyst stage [9]. In our study, the incidence of de novo segmental-chromosome abnormalities increased as the trophoblastic grade decreased, which was similar to previous studies [18, 56]. Whole-chromosome abnormalities are probably due to a high incidence of meiosis errors [54], which shows a strong association with increasing female age. Interestingly, we found that whole-chromosome abnormalities were related to biopsy days in the study. Faster-developing blastocysts were found to have a lower incidence of whole de novo chromosome abnormalities (biopsied on day 5 vs. day 6 vs. day 7: 10.4% vs. 15.6% vs. 18.8%, respectively; Figure 3). It can be speculated that blastocysts with faster development have a smaller maternal meiosis error rate. In addition, we found that complex chromosome abnormalities were associated with the poor quality and slower development of blastocysts. This result is consistent with the results of previous studies [55]. Embryos with de novo complex chromosome abnormalities usually suffer from the chaotic division of chromosomes [21] and undergo developmental arrest, significantly affecting embryo quality. Thus, poor-quality blastocysts are more prone to complex chromosome abnormalities. It is potentially useful to judge embryos with a high risk of complex chromosome abnormalities.

The main limitations of the study are twofold. First, it was a retrospective analysis. Second, patients who underwent PGT-A may be of advanced age, have repeated implantation failure, or have a history of repeated abortion, which may lead to a biased or inaccurate result. The rigorous control group comprised couples undergoing age-matched genetic testing for monogenic disorders (PGT-M) in the same period. The PGT-M patients did not have an infertility problem, and PGT-M did not increase the risk of aneuploidy in their embryos. However, the sample size of PGD-M patients at our center was too small, so more prospective studies with larger cohorts are needed to confirm the current results in the future.

In conclusion, older maternal age leads to an increase in the incidence of de novo chromosome abnormalities. Poor blastocyst quality is related to a higher incidence of de novo chromosome abnormalities in our study. Although morphologic evaluation cannot ensure chromosomally normal embryos, the study might provide some important information for selecting embryos for non-PGT patients. Transferring blastocysts with high morphologic scores is helpful to reduce de novo chromosome abnormality risk and implantation failure in particular. A significant proportion of aneuploid embryos are capable of achieving the highest scores, and some euploid embryos are morphologically poor. However, morphological evaluations cannot replace invasive embryo biopsy. This remains the only effective means of detecting aneuploidy at the blastocyst stage.

## Figures and Tables

**Table 1:** Basic data of PGT cycles in the study.

Basic clinical data	Description	
	PGT-SR cycles	PGT-A cycles
Number of cycles, *n*	283	173
Mean female age (years, ±SD)	29.7 ± 4.4	34.9 ± 4.7^∗∗^
Mean male age (years, ±SD)	30.5 ± 4.6	35.6 ± 6.2^∗∗^
Mean number of retrieved oocytes	17.8 ± 8.4	14.4 ± 8.1
2PN fertilized oocytes, *n* (%)	3,117 (78.6%)	1,569 (80.1%)
Available day 3 embryos obtained (%)	2,483 (48.9%)	1,276 (81.3%)^∗∗^
Available blastocysts, *n* (%)	1,213 (38.9%)	675 (43.0%)^∗∗^
Blastocysts for PGT analysis, *n*	1,160	604
Blastocysts with genetic results, *n* (%)	1,135 (97.8%)	590 (97.7%)
Blastocysts with no results, *n* (%)	25 (2.2%)	14 (2.3%)
Euploid blastocysts, *n* (%)	410 (36.1%)	297 (50.3%)^∗∗^
Aneuploid blastocysts, *n* (%)	639 (56.3%)	214 (36.3%)^∗∗^
Mosaic blastocysts, *n* (%)	86 (7.6%)	79 (13.4%)^∗∗^
De novo chromosome abnormalities, *n* (%)	318 (28.0%)	214 (36.3%)^∗∗^
De novo whole-chromosome abnormality, *n* (%)	112 (9.9%)	125 (21.2%)^∗∗^
De novo segmental-chromosome abnormality, *n* (%)	143 (12.6%)	53 (9.0%)^∗^
De novo complex chromosome abnormality, *n* (%)	63 (5.6%)	36 (6.1%)

*Note*. Values are presented as numbers, *n* (%). ^*∗*^*P* < 0.05, there was a significant difference compared with PGT-SR.  ^*∗∗*^*P* < 0.01, there was a significant difference compared with PGT-SR.

**Table 2:** Incidence of de novo chromosome abnormalities at different ages.

Maternal age	Incidence of de novo chromosome abnormalities		Total^1^
	PGT-SR cycles	PGT-A cycles	
≤29	24.6% (148/602)	21.6% (29/134)	24.0% (177/736)
30–34	30.7% (134/436)	32.8% (84/256)	31.5% (218/692)
35–39	34.9% (29/83)	42.3% (58/137)	39.5% (87/220)
≥40	50.0% (7/14)	68.3% (43/63)	64.9% (50/77)
Total^2^	28.0% (318/1,135)	36.3% (214/590)^a^	30.8% (532/1,725)
*χ* ^2^	10.071	43.790	65.840
*P*	0.018^∗^	0.000^∗^	0.000^∗^

*Note*. Values are presented as numbers, *n* (%). ^a^There was a significant difference compared with PGT-SR. Total^1^ represents the sum of PGT-SR and PGT-A cycles. Total^2^ represents all patients at different ages. ^*∗*^*P* < 0.05 was considered statistically significant.

**Table 3:** Relationship between the incidence of de novo chromosome abnormalities and number of oocytes obtained.

No. of oocytes retrieved	Incidence of de novo chromosome abnormalities		Total^1^
	<35 years old	≥35 years old	
≤5	30.4% (7/23)	38.5% (5/13)	33.3% (12/36)
6–10	29.9% (35/117)	50.7% (38/75)	40.1% (73/192)
11–15	27.8% (110/395)	48.5% (48/99)	31.6% (158/494)
≥16	27.2% (243/893)	41.8% (46/110)	24.3% (289/1,003)
Total^2^	27.7% (395/1,428)	46.1% (137/297)	30.8% (532/1,725)
*χ* ^2^	0.482	1.973	6.981
*P*	0.923	0.578	0.073

*Note*. Values are presented as numbers, *n* (%). Total^1^ includes the younger and older age groups. Total^2^ represents all patients at different ages.

**Table 4:** Incidence of de novo chromosome abnormalities in relation to embryo quality in PGT cycles.

	De novo chromosome abnormality		OR	95% CI	*P*	OR^a^	95% CI^a^	*P* ^a^
	No	Yes						
D2 cell number								
No	332 (27.8%)	157 (29.5%)	1.084	0.866–1.356	0.481	1.079	0.859–1.355	0.515
Yes	861 (72.2%)	375 (70.5%)	Ref			Ref		
D3 cell number								
4-5 cells	67 (5.6%)	38 (7.1)	1.433	0.934–2.199	0.099	1.489	0.965–2.300	0.072
6-7 cells	263 (22.1%)	144 (27.1%)	1.232	0.857–1.552	0.215	1.248	0.896–1.739	0.191
9-10 cells	190 (15.9%)	85 (16.0%)	1.153	0.857–1.552	0.347	1.135	0.840–1.535	0.409
≥11 cells	95 (8.0%)	43 (8.1%)	1.185	0.802–1.751	0.395	1.178	0.792–1.754	0.419
8 cells	578 (48.4%)	222 (41.7%)	Ref			Ref		
D3 fragmentation rate								
≤5	442 (37.0%)	171 (32.1%)	0.510	0.250–1.039	0.064	0.501	0.242–1.035	0.062
6–20	518 (43.4%)	243 (45.7%)	0.624	0.308–1.265	0.191	0.629	0.306–1.293	0.207
21–49	212 (17.8%)	99 (18.6%)	0.620	0.299–1.286	0.199	0.591	0.281–1.244	0.166
≥50	21 (1.8%)	14 (2.6%)	Ref			Ref		
Day of biopsy								
Day 5 biopsy	512 (42.9%)	170 (32.0%)	0.453	0.300–0.684	0.000	0.452	0.297–0.686	0.000
Day 6 biopsy	617 (51.7%)	314 (59.0%)	0.693	0.466–1.032	0.071	0.678	0.452–1.017	0.060
Day 7 biopsy	64 (5.4%)	48 (9.0%)	Ref			Ref		
Not evaluable ICM quality								
Grade A TE	124 (10.4%)	27 (5.1%)	0.373	0.241–0.576	0.000	0.375	0.241–0.582	0.000
Grade B TE	451 (37.8%)	141 (26.5%)	0.530	0.422–0.666	0.000	0.510	0.404–0.644	0.000
Grade C TE	618 (51.8%)	364 (68.4%)	Ref			Ref		
Not evaluable TE quality								
Grade A ICM	163 (13.7%)	46 (8.6%)	0.581	0.411–0.821	0.002	0.573	0.404–0.813	0.002
Grade B ICM	1,030 (86.3%)	486 (91.4%)	Ref			Ref		
Degree of the blastocyst expansion								
EXP3	3 (0.2)	3 (0.5)	2.943	0.618–14.002	0.175	3.258	0.670–15.831	0.143
EXP4	1,077 (90.3%)	476 (89.5%)	0.942	0.600–1.480	0.795	0.945	0.595–1.500	0.810
EXP5	51 (4.3%)	24 (4.5%)	1.039	0.540–1.998	0.910	1.080	0.554–2.106	0.821
EXP6	62 (5.2%)	29 (5.5%)	Ref			Ref		

*Note*. Values are presented as numbers, *n* (%). Ref: reference group. The *P* value was calculated using a univariable mixed logistic model. ^a^OR adj: odds ratios were adjusted according to female age and PGT-SR/PGT-A scheme. ^a^*P* value was calculated using a mixed logistic model adjusted according to female age and PGT-SR/PGT-A scheme.

## Data Availability

The data used during the current study are available from the corresponding author on reasonable request.
